# Arsenite-Induced Pseudo-Hypoxia Results in Loss of Anchorage-Dependent Growth in BEAS-2B Pulmonary Epithelial Cells

**DOI:** 10.1371/journal.pone.0114549

**Published:** 2014-12-16

**Authors:** Fei Zhao, Scott W. Malm, Alyssa N. Hinchman, Hui Li, Connor G. Beeks, Walter T. Klimecki

**Affiliations:** Department of Pharmacology and Toxicology, College of Pharmacy, University of Arizona, Tucson, Arizona, United States of America; New York University School of Medicine, United States of America

## Abstract

Epidemiology studies have established a strong link between lung cancer and arsenic exposure. Currently, the role of disturbed cellular energy metabolism in carcinogenesis is a focus of scientific interest. Hypoxia inducible factor-1 alpha (HIF-1A) is a key regulator of energy metabolism, and it has been found to accumulate during arsenite exposure under oxygen-replete conditions. We modeled arsenic-exposed human pulmonary epithelial cells *in vitro* with BEAS-2B, a non-malignant lung epithelial cell line. Constant exposure to 1 µM arsenite (As) resulted in the early loss of anchorage-dependent growth, measured by soft agar colony formation, beginning at 6 weeks of exposure. This arsenite exposure resulted in HIF-1A accumulation and increased glycolysis, similar to the physiologic response to hypoxia, but in this case under oxygen-replete conditions. This “pseudo-hypoxia” response was necessary for the maximal acquisition of anchorage-independent growth in arsenite-exposed BEAS-2B. The HIF-1A accumulation and induction in glycolysis was sustained throughout a 52 week course of arsenite exposure in BEAS-2B. There was a time-dependent increase in anchorage-independent growth during the exposure to arsenite. When HIF-1A expression was stably suppressed, arsenite-induced glycolysis was abrogated, and the anchorage-independent growth was reduced. These findings establish that arsenite exerts a hypoxia-mimetic effect, which plays an important role in the subsequent gain of malignancy-associated phenotypes.

## Introduction

Inorganic arsenic is unique among environmental toxicants in several ways. Epidemiological research has established it as an unequivocal human carcinogen, but there is no consensus as to its carcinogenic mechanism of action. Diseases and tissues targeted by arsenic are unprecedented in their diversity, including cancer and chronic non-cancer diseases targeting several tissues. Among these targets is the lung, an organ in which studies have established a strong link between environmental arsenic exposure and cancer, including (epithelial) squamous cell, adenocarcinoma and small cell sub-types [1]–[4]. The unparalleled diversity of pathologies caused by arsenic could be due to a small number of fundamental biological processes that are disrupted, resulting in a context-dependent set of pathologies in target tissues. We have previously shown that arsenite (As), a prototypical inorganic arsenic form, perturbs one such fundamental process, energy metabolism [5].

Glycolysis is the first stage of glucose metabolism. This non-oxygen-dependent process involves the conversion of cytosolic glucose to pyruvate in a sequence of ten cytosolic, enzyme-catalyzed reactions, with a net yield of two adenosine tri-phosphate (ATP) molecules. Under oxygen-sufficient conditions within the mitochondria, pyruvate is converted to acetyl-coenzyme A (Acetyl-CoA), which can then enter the tricarboxylic acid cycle (TCA). Reduced nicotinamide adenine dinucleotide (NADH) and succinate generated by the TCA cycle are then utilized by oxidative phosphorylation to produce 36 ATP molecules per molecule of glucose. Malignantly transformed cells commonly shift ATP production from oxidative phosphorylation to glycolysis, even under oxygen-replete conditions [6]–[8]. This “aerobic glycolysis”, also known as the “Warburg effect”, seems paradoxical given the comparatively inefficient production of ATP by glycolysis. Nevertheless, the shift to glycolysis is advantageous for proliferative tissue [9]. Glycolysis has a higher turnover rate than oxidative phosphorylation, and can sustain a high rate of ATP production [10]. Intermediates from glycolysis can serve as precursors for key macromolecules needed to support proliferation. Glucose-6-phosphate, fructose-6-phosphate, and glyceraldehyde-3-phosphate contribute to the production of ribose-5-phosphate, which can be used in nucleotide synthesis [11]. Amino acid synthesis can also utilize glycolysis intermediates. Pyruvate can serve as a precursor to alanine, valine, and leucine; 3-phospho-glycerate can be a precursor to serine, cysteine, and glycine [12].

Hypoxia inducible factor-1 alpha (HIF-1A) is a transcription factor controlling the expression of a battery of genes that regulate cellular processes that include glycolysis [13], [14]. Under oxygen sufficient conditions, HIF-1A is under tight regulation by prolyl hydroxylase domain (PHD) proteins and von Hippel-Lindau (VHL) protein. PHD hydroxylates HIF-1A at proline-403, proline-56, or both, in a process that requires oxygen and α-ketoglutarate [15], [16]. Hydroxylation of HIF-1A enables the binding of VHL, which is the recognition subunit of an E3 ubiquitin ligase adapter that mediates poly-ubiquitylation and proteasomal degradation of HIF-1A [17]. When oxygen is limited, PHD cannot hydroxylate HIF-1A, resulting in attenuated HIF-1A interactions with VHL. In this way HIF-1A is stabilized, and available to heterodimerize with constitutively expressed hypoxia inducible factor-1 beta (HIF-1B) to activate the transcription of target genes. HIF-1A protein can also be stabilized through non-oxygen dependent processes through mechanisms that are poorly understood. In particular, exposure to metals, including arsenite, can result in accumulation of HIF-1A [5], [18], [19].

The ability of arsenite to increase HIF-1A and glycolysis in an *in vitro* model of pulmonary epithelium generated interest as to whether these effects could be related to arsenite-induced malignant transformation in the lung. We tested one aspect of this in the BEAS-2B cell line, an *in vitro* model that has been successfully used in studies of arsenite-induced malignancy [20]–[23].

## Materials and Methods

### Reagents

Sodium arsenite 50 mM stock solution and MG132 were purchased from Sigma-Aldrich (St. Louis, MO).

### Cell culture

BEAS-2B (ATCC, Manassas, VA) is an SV40 immortalized, non-malignant cell line isolated from normal human bronchial epithelium [24]. The identity of BEAS-2B cells in culture was confirmed by genotyping using short tandem repeat analysis (CODIS identity markers) of nuclear DNA. BEAS-2B cells used in this study were tested monthly for mycoplasma contamination and remained mycoplasma-negative throughout the study. BEAS-2B was cultured in defined BEGM media (Lonza, Walkersville, MD). Two million cells were seeded to 75 cm^2^ culture flasks and subcultured when 90% confluence was reached. Trypsin-EDTA (0.25%) was used to remove cells from culture flasks for sub-culturing. All cells were incubated under 5% CO_2_ at 37°C during culture.

### Arsenite exposure

Cells were exposed to arsenite (1 µM final concentration) in culture media continuously for durations indicated in each experiment. Media additions between sub-culturing were maintained at 1 µM arsenite. Media replacement at sub-culturing was also maintained at 1 µM arsenite.

### Establishment of stable genetically modified derivative cell lines

Control and HIF-1A shRNA lentiviral particles were purchased from Santa Cruz Biotechnology. BEAS-2B cells were infected with control and HIF-1A shRNA lentiviral particles at an MOI (multiplicity of infection) of 10. Forty-eight hours after infection, cells were selected for 2 weeks (3 µg/mL puromycin in BEGM media).

### Lactate measurement

L-lactate levels were measured in culture media using the L-lactate assay kit (Biomedical Research Service Center, Buffalo, NY) according to manufacturer protocol. Forty-eight hours prior to analysis, cells were transferred to 35 mm cell culture dishes at identical density to minimize potential variability introduced by cell culture density; 4 hours prior to analysis, culture media was replaced with 1 mL of fresh culture media. For extracellular lactate determination, 800 µL of supernatant media was collected directly from the culture. Samples were deproteinized by 25% w/v polyethylene glycol PEG-8000 precipitation and clarified by centrifugation at 20,000 g for 5 min. The accuracy of lactate measurements was verified previously by inter-lab comparison with duplicate samples analyzed by the Comparative Pathology Laboratory, University of California, Davis [5].

### Antibodies and immunoblot analysis

Primary antibodies were used at the following dilutions: hypoxia-inducible factor 1 alpha (HIF-1A) 1∶250, E-cadherin 1∶250, and α-tubulin 1∶1000 (Santa Cruz Biotechnology, Santa Cruz, CA); goat anti-rabbit IgG-HRP and goat anti-mouse IgG-HRP (Santa Cruz Biotechnology) 1∶5000. Following experimental treatment, cells were washed twice with PBS and lysed in sample buffer [10% glycerol, 100 mM DTT, 50 mM Tris-HCl (pH 6.8), 2% SDS]. Samples were then denatured at 90°C for 5 min. After sonication, protein concentration was measured (Pierce 660 nm Protein Assay, Thermo Scientific, Rockford, IL). Equal protein masses from samples in sample buffer (final concentration 0.1% v/w bromophenol blue) were subjected to SDS-polyacrylamide gel electrophoresis and immunoblot analysis. Immunoblots were visualized by chemiluminescence (Thermo Scientific, Rockford, IL) and quantified using a GeneGenome5 imaging system (Syngene, Frederick, MD).

### RNA isolation and quantification

Total RNA was isolated from BEAS-2B cells using the RNeasy Mini Kit (Qiagen, Valencia, CA) according to manufacturer protocol. Quantitative PCR oligonucleotides (Life Technologies, Grand Island, NY) were HIF-1A (Hs00153153_m1) and GAPDH (HS 099999905_m1). Quantitative real-time PCR (QPCR) was performed with TaqMan One-Step RT-PCR Master Mix (Life Technologies, Grand Island, NY) according to manufacturer protocol using the StepOnePlus Real-Time PCR system (Life Technologies).

### Soft agar colony formation assay

Cells were removed from culture flasks with trypsin, suspended in culture media, and used in soft agar assays to measure anchorage-independent growth. Two mL of 0.7% agar in complete growth media was used to cover the bottom of each well (6-well plate). Ten thousand cells were suspended in 2 mL of 0.35% agar in complete growth media and overlaid onto base agar. Each agar layer was allowed to solidify for 30 min at room temperature. Two mL of BEGM media was placed over the agar layers, and was replaced with fresh media every 3 days. After 14 days of incubation, agar plates were stained for 8 hours with MTT (Sigma-Aldrich) to identify viable colonies. Plates were digitally photographed at identical exposure settings under fluorescent transillumination. Digital images were analyzed with identical analysis parameters using the particle count module of NIH ImageJ in order to enumerate the number of viable colonies.

### Ploidy measurement

Cells were plated in 60 mm dishes at a density of 1 million cells per dish. When cells were 80–90% confluent, media was removed, cells were trypsinized, quenched with defined trypsin inhibitor (Life technologies), and were washed twice with PBS. Cells were centrifuged at 1000 g for 10 min at 4°C. PBS was removed, and cells were fixed by slowly adding 1 mL of ice-cold 70% ethanol while vortexing. Cells suspended in ethanol were stored at −20°C overnight. Prior to analysis, fixed cells were centrifuged at 1500 g for 15 min at 4°C, ethanol was removed, and cells were resuspended in 0.5 mL cold PBS containing a final concentration of 0.5 mg/mL RNAse A (Life Technologies, Grand Island, NY) and 0.04 mg/mL propidium iodide (Sigma Aldrich, St. Louis, MO). Samples were then incubated at 37°C for 30 min while protected from light. Samples were analyzed using a FACScan cytometer (BD Bioscience, San Jose, CA), at excitation/emission wavelengths of 488/650 nm. A total of 50,000 events were collected for each sample. Ploidy analysis was performed using ModFit 3.0 (Verity Software House, Topsham, ME).

### Transfection

Transfection was performed with 1 µg of DNA plasmid using the Invitrogen Neon system (Life Technologies) at the following parameters: Cell density 5×10^6^ cells/mL, pulse voltage 1290 V, pulse width 20 ms, pulse number 2. The plasmid used for transfection, HA-HIF-1A P402A/P564A-pcDNA3 (Addgene, Cambridge, MA) has been described [24]. After transfection, cells were transferred to a 6-well plate for 48 hours prior to use.

### HIF-1A protein half-life measurement

To measure the half-life of HIF-1A, cells were exposed to 1 µM sodium arsenite or vehicle control for 2 weeks. Cycloheximide (CHX, 50 µM final concentration) was added to block protein synthesis as previously described [25]. Cell lysates were collected at 0, 2.5, 5, and 10 minute time-points and processed for immunoblot analysis for HIF-1A as described above.

### Immunofluorescence staining

BEAS-2B cells were grown on collagen coated (Sigma Aldrich) glass coverslips in 6-well plates. Cells on coverslips were fixed in ice-cold methanol and incubated at −20°C for one hour. Coverslips were then washed in PBS and incubated in anti-HIF-1A primary antibody diluted 1∶100 in PBS containing 10% fetal bovine serum for 50 min. After primary antibody incubation, coverslips were washed in PBS followed by a 50 minute incubation in secondary antibody (Alexa Fluor 488-conjugated anti-rabbit IgG, Life Technologies) diluted 1∶100 in PBS containing 10% fetal bovine serum and DAPI (300 nM, Life Technologies). Finally, the coverslips were washed in PBS and mounted with ProLong Gold Antifade Reagent (Life Technologies) on glass slides. Stained cells were imaged using the 3i Marianas Ziess Observer Z1 system and Slidebook 5.0 (Intelligent Imaging Innovations, Denver, CO).

### Sub-cellular fractionation

Fractionation of BEAS-2B cells was performed using NE-PER nuclear and cytoplasmic extraction reagents according to manufacturer protocol (Thermo Scientific, Rockford, IL). Briefly, BEAS-2B cells were trypsinized, quenched with defined trypsin inhibitor, and washed with PBS. Five million cells from each treatment group were processed for isolation of nuclear and cytoplasmic fractions. Cytoplasmic (supernatant) and nuclear extracts were subjected to immunoblot analysis.

### Metabolomic analysis

#### Cell culture extraction

1 µM sodium arsenite-treated (two weeks) and control cells were trypsinized and washed twice with ice-cold PBS. Three biological replicates were analyzed for each group. Six million cells per sample were pelleted and snap frozen in liquid nitrogen to preserve their metabolic state. Pellets were submitted to the Metabolomics Core Facility (University of Utah) for GC-MS analysis. Briefly, proteins were removed by precipitation as previously described [26]. Three hundred and sixty µL of −20°C, 90% methanol (aq.) was added to 40 µL of the individual tubes containing the cell pellets to give a final concentration of 80% methanol. The samples were incubated for one hour at −20°C followed by centrifugation at 30,000 g for 10 min using a rotor chilled to −20°C. The supernatant containing the extracted metabolites was then transferred to fresh disposable tubes and completely dried by vacuum.

#### GC-MS analysis

All GC-MS analysis was performed with a Waters GCT Premier mass spectrometer fitted with an Agilent 6890 gas chromatograph and a Gerstel MPS2 autosampler. Dried samples were suspended in 40 µL of 40 mg/mL O-methoxylamine hydrochloride (MOX) in pyridine and incubated for one hour at 30°C. Twenty-five µL of this solution was transferred to autosampler vials. Ten µL of N-methyl-N-trimethylsilyltrifluoracetamide (MSTFA) was added automatically via the autosampler and incubated for 60 min at 37°C with shaking. After incubation, 3 µL of a fatty acid methyl ester standard was added via the autosampler then 1 µL of the prepared sample was injected into the gas chromatograph inlet in the split mode with the inlet temperature held at 250°C. A 5∶1 split ratio was used. The gas chromatograph had an initial temperature of 95°C for one minute followed by a 40°C/min ramp to 110°C and a hold time of 2 min. This was followed by a second 5°C/min ramp to 250°C, a third ramp to 350°C, then a final hold time of 3 min. A 30 m Phenomex ZB5-5 MSi column with a 5 m long guard column was employed for chromatographic separation. Helium was used as the carrier gas at 1 mL/min.

#### Analysis of GC-MS data

Data was collected using MassLynx 4.1 software (Waters). A targeted approach for known metabolites was used. These were identified and their peak area was recorded using QuanLynx. Metabolite identity was established using a combination of an in-house metabolite library developed using pure purchased standards and the commercially available NIST library.

### Cell proliferation

To measure the effect of arsenite on cell proliferation, cells were trypsinized and counted with a Scepter 2.0 automated cell counter (Millipore, Darmstadt, Germany). Cell population doubling time was determined with the following equation as previously described: D_1_ =  ((total days of growth (D)) × Log2/Log (# of cells Time _D_/# of cells Time _0_)) ×24 [27].

### Statistical analysis

For data containing two comparison groups, unpaired t-tests were used to compare mean differences between control and treatment groups at a significance threshold of P<0.05. For data containing three or more groups, univariate ANOVA analysis, followed by Tukey's post hoc test, was used to compare mean differences of groups at a significance threshold of P<0.05. GraphPad Prism version 6.0 for MAC (GraphPad, La Jolla, CA) was used for all statistical analysis.

## Results

### Arsenite mediated HIF-1A accumulation is consistent with protein stabilization

**Figure 1:**
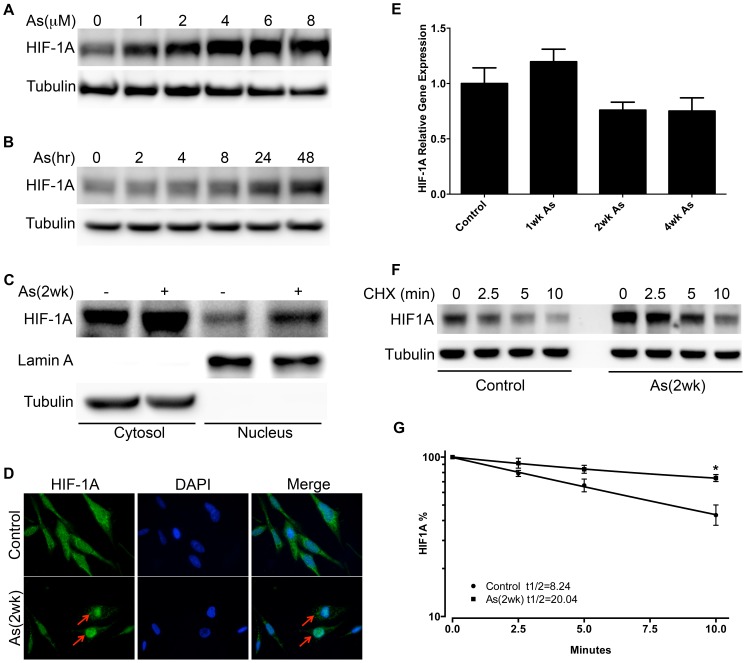
Arsenite causes HIF-1A accumulation/translocation in BEAS-2B. A) Immunoblot analysis of HIF-1A in BEAS-2B treated with 0–8 µM arsenite for 48 hours. B) Immunoblot analysis of HIF-1A in BEAS-2B treated with 1 µM arsenite for 0–48 hours. C) Immunoblot analysis of nuclear and cytosolic fractions of BEAS-2B, control or treated with 1 µM arsenite for 2 weeks, probed for HIF-1A, Lamin A (a nuclear marker) and tubulin (a cytosolic marker). D) Immunofluorescence staining of HIF-1A in BEAS-2B, control or treated with 1 µM arsenite for 2 weeks, arrows show HIF-1A nuclear accumulation. E) QPCR of HIF-1A mRNA in BEAS-2B treated with 1 µM arsenite for 0–4 weeks, bars represent mean, 1 standard deviation. F) Half-life measurement of HIF-1A in BEAS-2B, control or treated with 1 µM arsenite for 2 weeks, protein synthesis blocked with cycloheximide (CHX) for 0–10 min, followed by HIF-1A immunoblot. G) Quantification of HIF-1A protein half-life (t1/2). Densitometry of HIF-1A normalized to Tubulin was used for calculation. Points represent mean, +/− 1 standard deviation, 3 independent replicates. *p<0.05.

HIF-1A protein level was evaluated by immunoblot analysis, which revealed both time and dose-dependent arsenite-induced accumulation of HIF-1A (Fig. 1A, Fig. 1B). Functional transactivation by HIF-1A requires nuclear translocation. BEAS-2B exposed to 1 µM arsenite (2 weeks) showed increased accumulation of HIF-1A in both the nuclear and cytosolic fractions (Fig. 1C). Immunofluorescent staining confirmed accumulation of HIF-1A in the nucleus in arsenite-exposed BEAS-2B (Fig. 1D). To assess whether the accumulation of HIF-1A protein was due to its transcriptional up-regulation, BEAS-2B exposed to 1 µM arsenite (0, 1, 2, 4 week) were assayed by QPCR (Fig. 1E). No induction of HIF-1A at the transcriptional level was observed. Measurement of protein half-life, however, revealed that arsenite exposure resulted in a 43% increase in HIF-1A protein half-life (Fig. 1F, Fig. 1G), suggesting that accumulation of HIF-1A is due to protein stabilization.

### HIF-1A accumulation increases glycolysis in BEAS-2B

**Figure 2:**
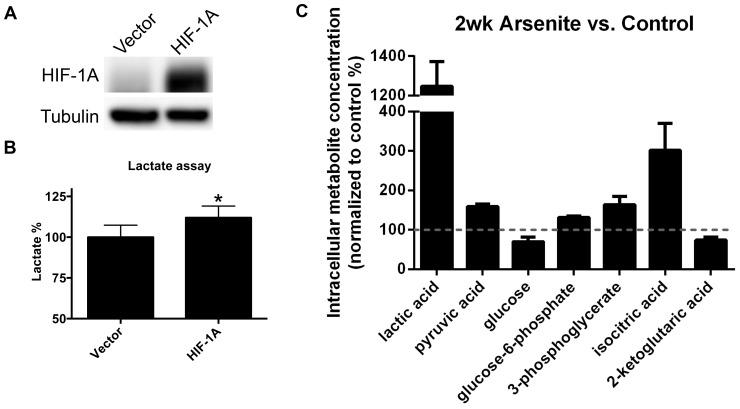
Glycolysis induction by HIF-1A overexpression in BEAS-2B. A) Immunoblot analysis of HIF-1A in BEAS-2B, vector control and transiently transfected with degradation-resistant HIF-1A mutant. B) Lactate levels (percent vector control) in cells described in 2A (Absolute lactate production in vector control: 0.729±0.054 µmol/10^6^cells/hr). Bars represent mean, 1 standard deviation, from 3 independent replicates. *p<0.05. C) Intracellular metabolite concentration (percent control BEAS-2B) of 1 µM arsenite-exposed (2 weeks) BEAS-2B cells. Bars represent mean, 1 standard deviation, from 4 experimental replicates. For each metabolite, levels in arsenite-exposed BEAS-2B are significantly different compared to control (p<0.05).

To evaluate the role of HIF-1A in arsenite-induced glycolysis in BEAS-2B, a degradation-resistant HIF-1A (HA-HIF-1A P402A/P564A) construct was transiently overexpressed in BEAS-2B (Fig. 2A) [28]. Lactate production in the HA-HIF-1A P402A/P564A expressing BEAS-2B was increased compared to vector transfected cells (Fig. 2B), suggesting that HIF-1A accumulation in BEAS-2B is sufficient to induce aerobic glycolysis. Metabolomic studies in control and 2 week arsenite exposed BEAS-2B revealed metabolite changes in the glycolytic pathway and TCA. In the arsenite-exposed BEAS-2B, lactic acid, pyruvic acid, glucose-6-phosphate 3-phosphoglycerate, and isocitric acid were found to be significantly increased compared to control. Glucose and 2-ketoglutaric acid (α-ketoglutarate) were decreased compared to control, consistent with the induction of glycolysis and suppression of the TCA cycle (Fig. 2C)

### HIF-1A-mediated glycolysis is associated with loss of anchorage-dependent growth in arsenite-exposed BEAS-2B

**Figure 3:**
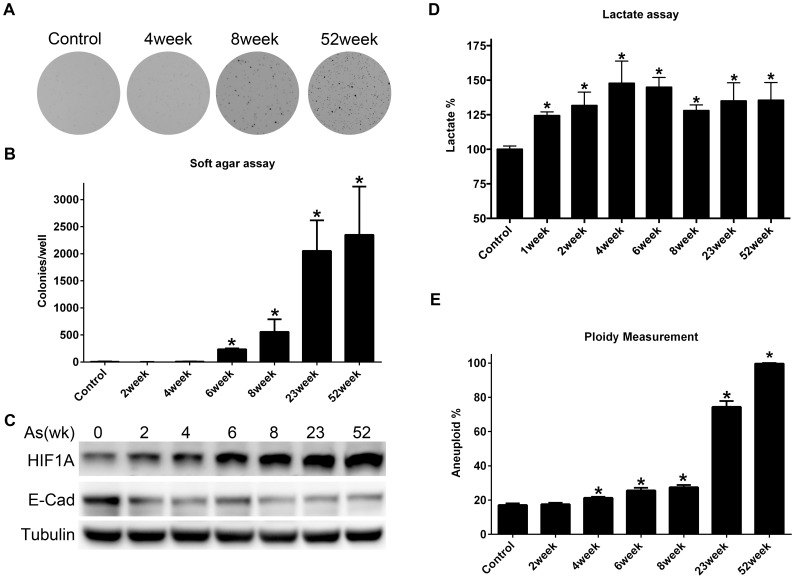
Arsenite-induced phenotypic changes in BEAS-2B. A) Representative images of soft agar growth over the course of 52 weeks of constant arsenite (1 µM) exposure. B) Colony counts in soft agar. Bars represent mean, 1 standard deviation, from 3 experimental replicates. C) Immunoblot analysis of HIF-1A and E-cadherin (E-cad) in BEAS-2B over the course of 52 weeks of constant arsenite (1 µM) exposure. D) Lactate levels (percent control) in BEAS-2B over the course of 52 weeks of constant arsenite (1 µM) exposure. Absolute lactate production in vector control: 0.733±0.017 µmol/10^6^cells/hr) Bars represent mean +1 standard deviation, from 3 experimental replicates. E) Percentage aneuploid cells in BEAS-2B treated with 1 µM arsenite for 0–52 weeks. Bars represent mean, +1 standard deviation, from 3 experimental replicates. *p<0.05.

Chronic exposure of BEAS-2B cells to 1 µM arsenite has been reported to malignantly transform BEAS-2B [29]. In this study, BEAS-2B acquired anchorage-independent growth at 6 weeks of arsenite exposure, and the ability to form colonies in soft agar further increased during continued arsenite exposure (Fig. 3A, Fig. 3B). Interestingly, aerobic glycolysis and accumulation of HIF-1A were observed at the earliest measurements (1 and 2 weeks) during the 52 weeks of arsenite exposure (Fig. 3C, Fig. 3D). This early response was also true for the loss of the epithelial identity marker, E-cadherin, which was substantially reduced at 2 weeks of arsenite exposure (Fig. 3C). The acquisition of aneuploidy, another marker of oncogenic transformation indicating substantial genome disruption associated with malignancy, did not rise substantially until later, between 8 and 23 weeks of arsenite exposure (Fig. 3E). From the initiation of arsenite exposure until the onset of soft agar growth (6 week arsenite exposure) no change in proliferative rate of BEAS-2B was observed (data not shown).

### HIF-1A knockdown suppresses arsenite-induced glycolysis and growth in soft agar

**Figure 4:**
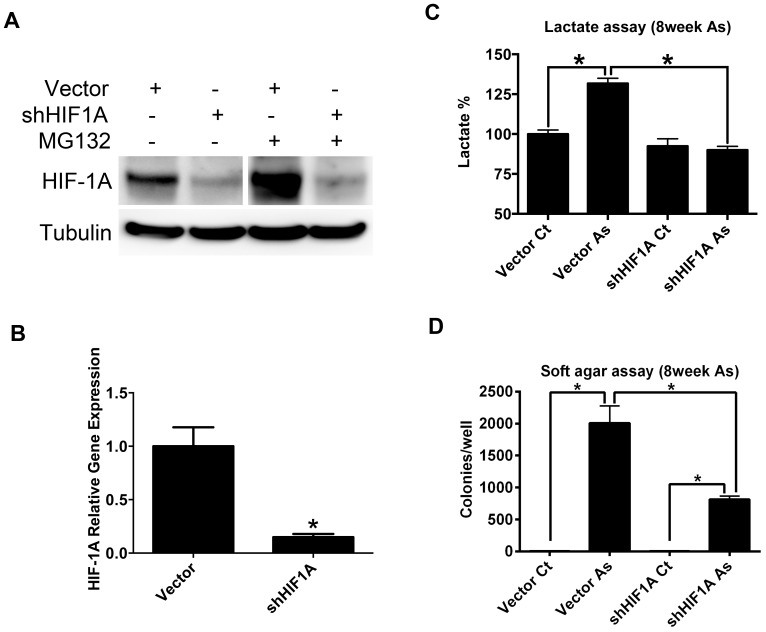
Effect of suppressed HIF-1A expression on arsenite mediated transformation. A) Immunoblot analysis of HIF-1A knockdown in BEAS-2B, short immunoblot exposure shown for MG132-treated samples; long immunoblot exposure shown for MG132-untreated samples. B) QPCR for HIF-1A mRNA. Bars represent mean, +1 standard deviation, from 5 experimental replicates. C) Lactate levels (percent control) in arsenite-exposed (denoted “As”, exposed for 8 weeks) and unexposed control (denoted “Ct”) BEAS-2B stably transfected with scrambled control shRNA (denoted “Vector”) or with shRNA targeting HIF1A (denoted “shHIF1A”) expression. Absolute lactate production in vector control: 0.696±0.04 µmol/10^6^cells/hr). Bars represent mean, +1 standard deviation, from 3 experimental replicates. D) Colony count of soft agar assay from BEAS-2B cells treated as described above in panel C. Bars represent mean, +1 standard deviation, from 3 experimental replicates. *p<0.05.

In order to understand the role of arsenite-induced glycolysis and HIF-1A stabilization in arsenite-mediated acquisition of malignancy-associated phenotypes, variants of the BEAS-2B cell line were developed that stably expressed empty lentiviral vector or shRNA targeting HIF-1A (shHIF1A). Both HIF-1A mRNA and protein levels were effectively suppressed by shHIF1A in BEAS-2B (Fig. 4A, Fig. 4B). Compared to shRNA scramble controls, the additional lactate production resulting from arsenite exposure was abrogated in BEAS-2B stably expressing shHIF1A (Fig. 4C), strongly suggesting that HIF-1A is essential to the induction of glycolysis by arsenite. At 8 weeks of arsenite exposure, blocking glycolysis and HIF-1A expression suppressed the acquisition of anchorage-independent growth resulting from arsenite exposure by about 50% (Fig. 4D).

## Discussion

BEAS-2B cells continuously exposed to 1 µM (75 ppb) arsenite in culture acquire anchorage-independent growth, a defining characteristic of malignant transformation. This is an effect of arsenite exposure that is consistent with other reports [22], [29]–[31]. We did not test arsenite-exposed BEAS-2B for *in vivo* xenograft formation, a complementary assay for malignant transformation. Thus, it is possible that the loss of anchorage-dependent growth in observed in our study may not correlate with in vivo malignancy. However, arsenite-induced growth in soft agar has been shown in other studies, including studies of arsenite-exposed BEAS-2B cells, to be associated with tumor formation in immunocompromised rodent models [22], [29].

In contrast to some published studies that demonstrated arsenite-induced transformation of BEAS-2B, work in this study used defined culture media (BEGM) that did not contain bovine serum. In a separate study (currently submitted for review), we report substantial phenotypic differences, including large-scale gene expression re-programming, induced by the presence of bovine serum in BEAS-2B culture medium. Thus, culture conditions used in studies of chemical carcinogenesis in BEAS-2B are an important consideration when comparing studies. We observed the first evidence of anchorage-independent growth in soft agar at 6 weeks of arsenite exposure, which is earlier than reported BEAS-2B studies of arsenite-induced malignant transformation, in which anchorage-independent growth was reported at exposure durations ranging from 16-26 weeks [22], [29], [32]. Our study represents the most rapid acquisition of a malignancy-related phenotype caused by inorganic arsenic exposure that we are aware of. Loss of anchorage dependence was not associated with loss of diploid genome content (Fig. 3E). At more extended durations of arsenite exposure, we did observe loss of control over genome content, as the proportion of tetraploid BEAS-2B cells increased substantially at 23 weeks of arsenite exposure. This suggests that exposure duration is another important consideration in evaluating *in vitro* malignant transformation by arsenite, since later events may be additionally impacted as a result of grossly disrupted genome content. Arsenite-induced soft agar growth was associated with an early loss of a biomarker of epithelial identity, E-cadherin. We did not observe an associated increase in mesenchymal markers (alpha smooth muscle actin, vimentin, data not shown) that would suggest canonical epithelial to mesenchymal transformation (EMT). This is consistent with arsenite causing loss of differentiation or metaplasia, rather than a true EMT. Arsenite exposure in BEAS-2B also resulted in an early dysregulation of cellular energy metabolism, a novel effect of arsenite that we have previously reported to be associated with accumulation of HIF-1A and the induction of a battery of glycolysis-associated genes [5]. Interestingly, in the microarray study performed by Stueckle, comparing chronic arsenic trioxide exposed BEAS-2B to controls, energy metabolism pathways were found to be disrupted. These pathways included carbohydrate metabolism, which is consistent with our findings [22].

Arsenite exposure in BEAS-2B appears to produce a “hypoxia-mimetic” effect characterized by an early HIF-1A protein accumulation. Unlike HIF-1A activation by chronic hypoxia, where HIF-1A accumulation is transient, the arsenite-induced accumulation of HIF-1A is sustained throughout the course of 52 weeks of exposure [33], [34]. We found that HIF-1A mRNA levels were not altered during arsenite exposure, consistent with published reports [35]. Arsenite exposure did impact HIF-1A protein half-life in BEAS-2B, with over a two-fold increase observed. Thus, the arsenite-induced HIF-1A protein accumulation that we observed appears to be due to protein stabilization, a process that can be mediated by prolyl hydroxylase domain (PHD) proteins [15], [16]. Metabolic intermediates of glucose metabolism can inhibit PHD function, and we observed elevated levels of two established PHD-inhibitory metabolites, pyruvate and isocitrate [36]. In addition, the level of α-ketoglutarate, a cofactor required for PHD-dependent hydroxylation of HIF-1A, was reduced by arsenite in BEAS-2B. Taken together, it is possible that arsenite-induced HIF-1A accumulation is due to metabolite-related inhibition of PHD function.

HIF-1A protein level is critical to the induction of aerobic glycolysis by arsenite in BEAS-2B. Overexpression of HIF-1A in BEAS-2B was sufficient to increase lactate production, albeit to a lesser extent than that induced by chronic arsenite exposure. Arsenite could be exerting effects on other targets that amplify the effect of HIF-1A. Established examples of such targets include the pyruvate dehydrogenase complex and oxidative phosphorylation proteins [37], [38]. Suppressing HIF-1A expression using shRNA-expressing derivative BEAS-2B cell lines abrogated arsenite-induced aerobic glycolysis, underscoring the importance of HIF-1A to arsenite-induced glycolysis.

The sustained HIF-1A protein accumulation resulting from arsenite exposure was also essential for maximal soft agar growth in arsenite-exposed BEAS-2B. BEAS-2B stably knocked down for HIF-1A expression had less than half the soft agar colony formation compared to vector control cells exposed to arsenite for 8 weeks. One explanation of these data is that the early, HIF-1A-mediated consequence of arsenite exposure may be in creating a “malignancy-permissive” state, which may not be sufficient to cause malignant transformation, but may amplify the effect of other factors that induce transformation. This effect could include cytoprotection. Work by Ganapthy S. et al. showed that arsenite exposure induces HIF-1A in normal mouse tissue, and was protective against cytotoxicity [39]. Additional mechanisms through which HIF-1A could enable transformation include hypoxic resistance and the enhanced production of macromolecular precursors resulting from increased glycolysis [40], [41].

This work establishes that an early consequence of *in vitro* arsenic-induced phenotypic transformation involves an inappropriate “pseudo-hypoxia” response that results in metabolic dysregulation, and is essential for acquisition of a key characteristic of malignant transformation: loss of anchorage-dependent growth. Future work will be aimed at defining the individual contributions of two important, concurrent effects of elevated HIF-1A levels in arsenite-exposed BEAS-2B: transcriptional activation of HRE-regulated genes and the induction of glycolysis. In addition, many of the mechanisms of arsenite-induced dysregulation of HIF-1A could potentially apply as well to HIF-2A, a HIF family member also implicated in the acquisition of malignancy. Subsequent work should assess a possible role of HIF-2A in arsenite-induced loss of cellular growth control. The role of disrupted energy metabolism in carcinogenesis is a rapidly growing area of cancer research. HIF-1A dysregulation and associated metabolic perturbation are early, important effects of arsenite that are important to its carcinogenic potential. As such, our findings offer exciting new mechanistic explanations to the conundrum of arsenic carcinogenesis.
